# Cytomegalovirus-vectored COVID-19 vaccines elicit neutralizing antibodies against the SARS-CoV-2 Omicron variant (BA.2) in mice

**DOI:** 10.1128/spectrum.02463-23

**Published:** 2023-11-16

**Authors:** Jian Liu, Dabbu Kumar Jaijyan, Yanling Chen, Changcan Feng, Shaomin Yang, Zhenglong Xu, Nichun Zhan, Congming Hong, Shuxuan Li, Tong Cheng, Hua Zhu

**Affiliations:** 1 School of Biological Sciences and Biotechnology, Minnan Normal University, Zhangzhou, Fujian, China; 2 Department of Microbiology, Biochemistry and Molecular Genetics, Rutgers-New Jersey Medical School, Newark, New Jersey, USA; 3 Shenzhen Municipal Key Laboratory for Pain Medicine, Department of Pain Medicine, Huazhong University of Science and Technology Union Shenzhen Hospital, Shenzhen, Guangdong, China; 4 State Key Laboratory of Molecular Vaccinology and Molecular Diagnostics, National Institute of Diagnostics and Vaccine Development in Infectious Diseases, School of Life Sciences, Xiamen University, Xiamen, Fujian, China; Chinese Academy of Sciences Wuhan Institute of Virology, Wuhan, China

**Keywords:** cytomegalovirus, viral vector-based vaccine, immunogenicity evaluation, SARS-CoV-2, variant of concern

## Abstract

Viral vector vaccine represents one of the important research directions of novel vaccines. Viral vectors can efficiently deliver foreign genes and drive robust transgene expression *in vivo*. Generally, viral vector vaccines can achieve high immunogenicity and rapid response to a new pandemic. However, few types of viral vectors have been thoroughly evaluated for vaccine development, and the application of viral vectors is often limited by packaging capacity, vector immunogenicity, or pre-existing immunity. Cytomegalovirus (CMV) has been regarded as a promising novel vaccine vector because it has the advantages of high package capacity, good safety profiles, and the capacity to induce long-lasting immune responses. In this study, a luciferase-tagged reporter murine CMV (MCMV) was constructed, and the viral dissemination feature *in vivo* was studied. Furthermore, MCMVs expressing three structural proteins of SARS-CoV-2 were constructed and used to immunize mice. The level of binding antibodies and neutralizing antibodies against the original Wuhan strain and the Omicron variant (BA.2) was compared. Moreover, the CMV vector’s transmission capacity and pre-existing immunity’s influence on CMV-vectored vaccine efficacy were studied. The results showed that CMV vector could only cause transient systemic, but not persistent, infection. Immunization of CMV-vectored vaccines was able to elicit neutralizing antibodies against the Omicron variant (BA.2). However, the titer of neutralizing antibodies against BA.2 is much lower when compared with the original strain. The studies also showed that the CMV vectors would not cause unexpected viral transmission, and pre-existing immunity might impair the immunogenicity of subsequent CMV-vectored vaccines. Collectively, the CMV-vectored vaccine represents a promising approach to developing novel vaccines against epidemic pathogens.

**IMPORTANCE:**

Cytomegalovirus (CMV) has been used as a novel viral vector for vaccine development and gene therapy. Coronavirus disease 2019 is an infectious disease caused by the SARS-CoV-2 virus, which is highly mutable and is still circulating globally. The study showed that the CMV viral vector caused transient systemic infection and induced robust transgene expression *in vivo*. CMV vectors expressing different SARS-CoV-2 proteins were immunogenic and could elicit neutralizing antibodies against a highly mutated Omicron variant (BA.2). The expression level of receptor-binding domain (RBD) protein was higher than that of full-length S protein using CMV as a vaccine vector, and CMV vector expression RBD protein elicited higher RBD-binding and neutralizing antibodies. Moreover, the study showed that CMV-vectored vaccines would not cause unexpected viral transmission, and pre-existing immunity might impair the immunogenicity of subsequent CMV-vectored vaccines. These works provide meaningful insights for the development of a CMV-based vector vaccine platform and the prevention and control strategies for SARS-CoV-2 infection.

## INTRODUCTION

Vaccination is one of the most cost-effective means to fight against infectious diseases. Coronavirus disease 2019 (COVID-19) is the latest major global pandemic caused by the SARS-CoV-2 virus, which caused hundreds of millions of infections and more than 6 million deaths globally (1, 2). From January 2020 to May 2023, COVID-19 was declared a public health emergency of international concern by the World Health Organization. The development of various vaccines has contributed significantly to creating herd immunity against SARS-CoV-2 during the global pandemic. However, SARS-CoV-2 is still prevalent in many areas of the world and generates new mutations (3, 4). Humans are still facing the threat of COVID-19 or other emerging infectious diseases. Developing novel vaccines that can rapidly respond to emerging infectious diseases or highly mutable viruses is of great importance for human beings.

The viral vector-based vaccine is a novel strategy for vaccine development, and it offers the potential to revolutionize vaccine development. Viral vector-based vaccines can achieve rapid response to new emerging infectious diseases because it is relatively quick and easy to construct and evaluate viral vector-based vaccines against different pathogens based on an established platform. The development of viral vector vaccines has progressed rapidly in recent years. In the battle against COVID-19, the COVID-19 adenoviral vector-based vaccine was first assessed in clinical trials in China and has been authorized for emergency use in many countries (5, 6). A live-attenuated influenza virus vector-based SARS-CoV-2 vaccine was evaluated and approved for emergency use in China (7). However, some significant problems still need to be solved in viral vector vaccine development. Currently, few types of viral vectors have been thoroughly evaluated for vaccine development, and the application of viral vectors is often limited by packaging capacity, vector immunogenicity, or pre-existing immunity (8 – 10). Therefore, developing novel viral vectors is crucial to the research of viral vector vaccines.

The use of cytomegalovirus (CMV) as a viral vector has attracted much attention recently because of its unique advantages (11 – 14). CMV-vectored vaccines have been evaluated in the vaccine research of various diseases, including HIV, tuberculosis, tumors, and COVID-19 (14 – 17). The CMV-vectored vaccines showed high immunogenicity and a good safety profile in these evaluations; however, some critical considerations, including whether the virus of the CMV-vectored vaccine retains transmission capacity and how the pre-existing immunity against the CMV vector affects vaccine efficacy still need to be clarified. In this study, luciferase-tagged murine CMV was constructed and used to study the dissemination feature *in vivo*. Three different CMV-vectored COVID-19 vaccines were constructed and evaluated for immunogenicity, vaccination strategy, and level of neutralizing antibodies against both the original strain and a highly mutated Omicron variant (BA.2). Furthermore, the transmission capacity and how the pre-existing immunity against CMV vector affected the immunogenicity of subsequent CMV-vectored vaccines were evaluated.

## MATERIALS AND METHODS

### Cells, viruses, and antibodies

NIH-3T3 (#CRL-1688) and human embryonic kidney cell line 293T (#CRL-3216) were obtained from ATCC (Manassas, VA, USA). 293T stably expressing human angiotensin-converting enzyme 2 (293T-ACE2) was constructed in the study. All the above cells were maintained in Dulbecco’s Modified Eagle’s Medium ( Gibco BRL, Gaithersburg, MD, USA) containing 10% fetal bovine serum (ExCell Bio, Inc., Shanghai, China), 120 U/mL penicillin, and 100 U/mL streptomycin (Beyotine, Shanghai, China). Insect cell line Sf21 (#B82101; Gibco BRL, Gaithersburg, MD, USA), derived from *Spodoptera frugiperda*, was routinely maintained in CCM_3_ medium (Hyclone GE Healthcare, Piscataway, NJ, USA) containing 2% FBS, 120 U/mL penicillin, and 100 U/mL streptomycin.

The murine cytomegalovirus (MCMV) used in the study was derived from the Smith strain. The MCMV genome was cloned as a bacterial artificial chromosome (BAC) and maintained in a bacterial strain SW102 (18). Recombinant MCMV-expressing luciferase, SARS-CoV-2 full-length spike protein (S-full), receptor-binding domain (RBD), SARS-CoV-2 nucleocapsid protein (N), or Zika virus (ZIKV) full-length E protein (E-full) were constructed by homologous recombination in the SW102. All these viruses were recovered by transfection of the recombinant BACs into NIH-3T3 cells.

Western blot detection of SARS-CoV-2 S protein and RBD was performed with an anti-RBD monoclonal antibody (#67758-1-Ig; Proteintech, Wuhan, China). SARS-CoV-2 N protein was detected with an anti-nucleocapsid monoclonal rabbit antibody (#40143-R019; Sino Biological Inc., Beijing, China). β-actin was used as an internal reference and detected with a monoclonal mouse antibody (#66009-1-Ig; Proteintech, Wuhan, China). ZIKV E protein was detected with a homemade monoclonal antibody (19).

### Construction of recombinant murine cytomegaloviruses

In this study, recombinant MCMVs were constructed via the CRISPR-Cas9 system, as described with some modifications (20, 21). Bacterial strain SW102 harboring the MCMV genome was grown to an OD_600_ of 0.6. Then, the culture was heat-shocked at 42°C for 15 min before making electrocompetent cells. The galK cassette containing 50 bp homology to the MCMV IE2 locus (184,293 bp) was electroporated into the competent cells, and the bacteria were recovered in 1 mL Super Optimal broth with Catabolite repression (SOC) medium for 1 h at 32°C (22). The bacteria were washed twice in 1×M9 salts and plated on M63 minimal media plates with galactose, leucine, biotin, and chloramphenicol. A few colonies were streaked, and a PCR assay was performed to verify colonies with the galK cassette inserted into the target location (MCMV-IE2-galK). Plasmid pCas was then transformed into the bacteria-harboring MCMV-IE2-galK, and 10 mM arabinose was added to the culture 1 h before electrocompetent cells were made. Editing templates were constructed by fusing 500 bp homology to IE2 locus with target genes expression cassette [driven by a CMV promoter and terminated by bovine growth hormone poly-adenylation signal (BGH-polyA)]. The sgRNA recognizing the galK gene was cloned into pTarget (pTarget-galK). The editing template and pTarget-galK were co-electroporated into the competent cells harboring MCMV-IE2-galK and pCas. The bacteria were recovered in 1 mL SOC medium for 1 h at 32°C and plated on LB agar plates containing kanamycin, spectinomycin, and chloramphenicol. A few colonies were picked, and a PCR assay was performed to confirm that the galK cassette had been replaced by the target gene cassette.

### Indirect enzyme-linked immunosorbent assay

The binding IgG antibodies in the sera were measured by indirect enzyme-linked immunosorbent assay (ELISA) (23). Purified SARS-CoV-2 RBD (319–541 aa) protein, N protein, or Domain III of ZIKV E (E-DIII, 296–403 aa) diluted in coating buffer (0.05 mol/L sodium carbonate-bicarbonate, pH 9.6) was coated onto 96-well ELISA plates (100 ng/well) and incubated overnight at 4°C. The plates were blocked with saturation buffer (Wantai Biological Pharmacy Enterprise Co., Beijing, China) at 37°C for 2 h. The sera (started from 1:50 dilution) were fivefold serially diluted with sample diluent and added to each well (100 µL/well). After incubation at 37°C for 1 h, the plates were washed five times with washing buffer (PBST, 0.05% Tween-20 in PBS buffer), and the wells were added with horseradish peroxidase-conjugated goat anti-mouse (1:5,000 in enzyme diluent) or goat anti-human IgG antibodies (1:5,000 in enzyme diluent). The plates were then incubated at 37°C for 30 min and washed five times with washing buffer. The reaction was developed by 3, 3′, 5, 5′-tetramethylbenzidine substrate for 15 min and stopped with 2.1 M H_2_SO_4_. The optical density values at 450 nm (OD_450_) and 620 nm (OD_620_) were read on a microplate reader, and the value of OD_450_–OD_620_ was calculated.

### Luciferase assay

Two different *in vitro* luciferase assays were performed in the study. If cells need to be kept alive for continuous tracing of luciferase expression, 150 µg/mL D-luciferin was directly added to the medium, and the cells were incubated at 37°C for 10 min. The bioluminescent signals were collected using an IVIS Imaging System (Xenogen) following the manufacturer’s instructions (24). The end-point luciferase assay will be performed according to the manufacturer’s instructions (Promega) if the cells need not be kept alive. Cells were plated and cultured in white-flat 96-well cell culture plates before the infection of luciferase-tagged viruses. The medium was removed at detection, and 25 µL lysis buffer was added to each well. The plates were vortexed on a plate vortexer for 10 min to lyse the cells, and 100 µL luciferase assay reagent was added to each well. The bioluminescent signals were collected with a SparkControl Magellan plate reader (Tecan, Männedorf, Switzerland).

An *in vivo* luciferase assay was performed to quantitatively trace the dissemination of MCMV-Luc in mice (24). MCMV-Luc-infected mice were injected intraperitoneally (i.p.) with 1.5 mg D-luciferin, and the mice were anesthetized with isoflurane inhalation. The bioluminescent signals were recorded 10 min post-D-luciferin injection using the IVIS imaging systems.

### Animal studies

In the experiments of tracing the CMV dissemination *in vivo*, female BALB/c mice (*n* = 3) were i.p. infected with either MCMV-Luc or MCMV-WT at a dose of 10^7^ median tissue culture infectious dose (TCID_50_) per mouse, and *in vivo* luciferase assay was performed every 2-4 days to record the bioluminescent signals. In the MCMV-WT infection group, mice were re-infected with MCMV-Luc (i.p.; 10^7^ TCID_50_), and the bioluminescent signals were recorded every 2-4 days. In the experiments of CMV-vectored vaccine immunization, three groups of female BALB/c mice (*n* = 4) were immunized with recombinant MCMVs expressing SARS-CoV-2 full-length spike (S) protein (MCMV-S-full), receptor-binding domain (MCMV-RBD), or nucleocapsid (MCMV-N) (i.p.; 10^7^ TCID_50_) at weeks 0, 4, and 8, and unimmunized mice (*n* = 2) were caged with immunized mice in all three groups. Blood was collected for antibody evaluation at weeks 0, 2, 4, 6, 8, 10, and 12. At week 12, MCMV-RBD immunized mice were cross-immunized with one dose of MCMV-N (i.p.; 10^7^ TCID_50_); MCMV-N immunized mice were cross-immunized with one dose of MCMV-RBD (i.p.; 10^7^ TCID_50_); MCMV-S-full immunized mice were cross-immunized with one dose of MCMV-Zika-E-full (i.p.; 10^7^ TCID_50_); unimmunized mice were immunized with one dose of MCMV-Zika-E-full (i.p.; 10^7^ TCID_50_). Bloods were collected at weeks 1, 2, and 3 post-cross-immunization.

### Neutralization assay

The level of neutralizing antibodies in mice sera was evaluated by neutralization assay (25). The volume of MCMV-Luc or luciferase-tagged pseudovirus sufficient to achieve 10^5^ RLUs of luciferase signal per well was determined. The mice sera were threefold serially diluted (started from 1:10 dilution) and incubated with reporter virus at 37°C for 1 h, and the mixtures were used to infect 3T3 cells or 293T-ACE2 cells. At 2.5 days (3T3 cells infected with MCMV-Luc) or 4 days (293T-ACE2 cells infected with luciferase-tagged pseudovirus) post-infection, *in vitro* luciferase assay (end-point method) was performed to measure the bioluminescent signals with a SparkControl Magellan plate reader (Tecan, Männedorf, Switzerland). The inhibition rate at each dilution of sera was calculated, and the 50% neutralization titer (NT50) was determined using the half-maximal inhibitory concentration values of the sera.

### Statistical analysis

Statistical analyses were performed in SPSS Statistics using the two-tailed Student *t*-test for two groups (26). *P < 0.05; **P < 0.01; and ***P < 0.001.

## RESULTS

### Luciferase-tagged murine cytomegalovirus caused transient systemic viral infection in mice

**Fig 1:**
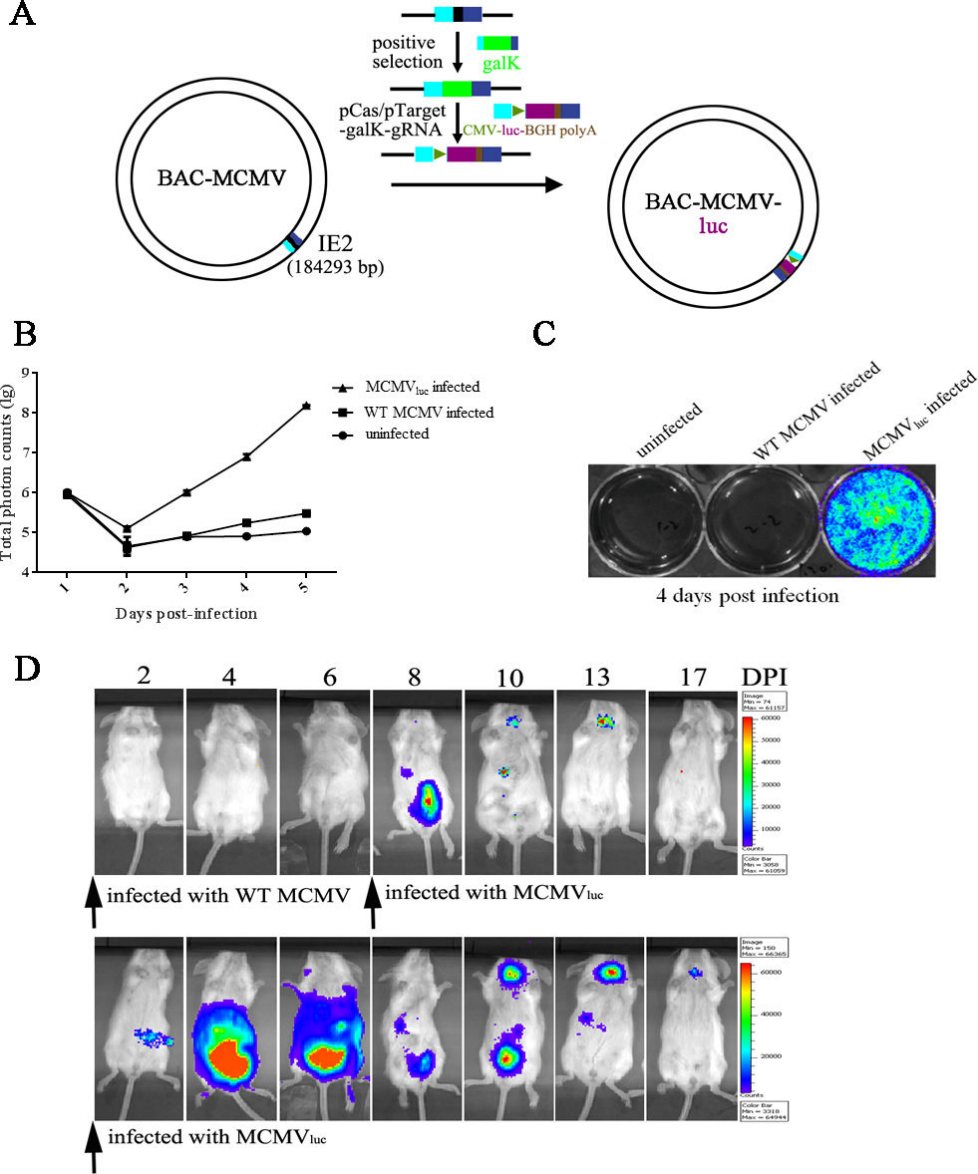
Construction of a luciferase-tagged MCMV and *in vivo* tracking of its dissemination. (**A**) Strategy for constructing MCMV-Luc. The luciferase expression cassette was inserted into the IE2 locus of MCMV via a modified CRISPR-Cas9 gene editing technology. (**B**) *In vitro* monitoring of virus growth in 3T3 cells with luciferase as a reporter. MCMV-Luc was used to infect 3T3 cells, and an *in vitro* luciferase assay was performed every day post-infection. MCMV-WT infection and mock infection were used as controls. (**C**) The bioluminescent signals were detected 4 days post-infection of mock, MCMV-WT, and MCMV-Luc using an IVIS Imaging System (Xenogen). (**D**) *In vivo* tracking of MCMV dissemination in mice. MCMV-Luc and/or MCMV-WT were used to infect mice (i.p.; 10^7^ TCID_50_), and the bioluminescent signals were detected every 2 days using an *in vivo* luciferase assay.

To quantitatively trace the virus replication *in vitro* and *in vivo*, luciferase-tagged MCMV (MCMV-Luc) was constructed. The firefly luciferase gene’s expression cassette under the CMV promoter’s control was constructed into the IE2 locus of the MCMV genome by homologous recombination in bacterial strain SW102 (Fig. 1A). The recombinant MCMV was recovered, and the luciferase expression was verified by cell-based luciferase assay. Compared with mock infection or wild-type MCMV (MCMV-WT) infection, increasing bioluminescent signals could be detected post-infection when using MCMV-Luc to infect NIH 3T3 cells. In contrast, no signal or only background signals were detected in the control groups (Fig. 1B and C).

To elucidate the CMV dissemination feature *in vivo*, MCMV-Luc and MCMV-WT were used to infect BALB/c mice intraperitoneally at a dose of 10^7^ TCID_50_ per mouse, and *in vivo* luciferase assays were conducted to monitor the replication of the virus. Weak bioluminescent signals could be detected at the injection site as early as 2 days post-infection in the MCMV-Luc infection group, and the signal increased rapidly to the peak and spread to the whole enterocoelia at 4 or 6 days post-infection. The bioluminescent signals at enterocoelia decreased, and the signals at the salivary gland appeared 10 days post-infection; it indicated that the virus was circulated to the salivary gland and started replicating there. The bioluminescent signal almost cannot be detected after 17 days post-infection. In the group of mice infected with MCMV-WT, no signal could be detected 2–6 days post-infection; when these mice were re-infected with MCMV-Luc at 6 days post-infection, bioluminescent signals could be detected at a much lower level after 2 days, and the virus disseminated in the same way as MCMV-Luc infected group (Fig. 1D). These results showed that MCMV would cause transient systemic infection and drive foreign gene expression in mice, and no serious health problem was observed in the infected mice.

### CMV-vectored COVID-19 vaccines elicited neutralizing antibodies against both the original strain and the BA.2 variant

**Fig 2:**
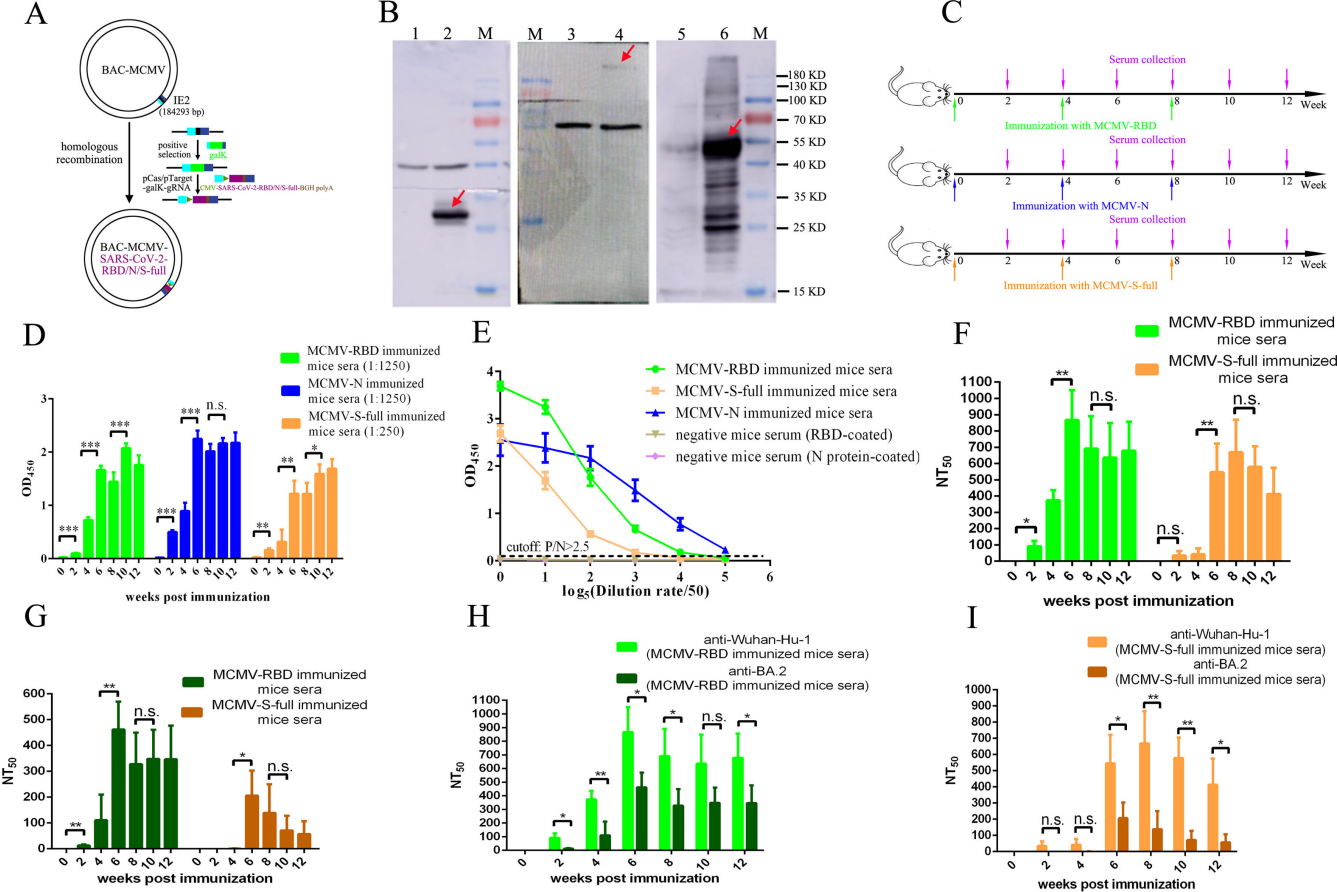
Evaluation of the immunogenicity of CMV-vectored vaccine expressing SARS-CoV-2 key constituent proteins. (**A**) Strategy for constructing MCMV-RBD/N/S-full. The RBD/N/S-full protein expression cassettes were inserted into the IE2 locus of MCMV via a modified CRISPR-Cas9 gene editing technology. (**B**) Detection of target gene expression in MCMV-RBD/N/S-full infected cells using Western blot. MCMV-RBD/N/S-full infected and uninfected cells were lysed, and the proteins were separated by SDS-PAGE and transferred to PVDF membranes. The expression of RBD (lane 2) or S-full (lane 4) in the infected cell lysates was probed with a monoclonal mouse antibody specific to RBD, and N protein (lane 6) was probed with a monoclonal rabbit antibody. Uninfected cells (lanes 1, 3, and 5) were set as control, and Beta-actin was used as an endogenous reference. (**C**) Schematic diagram of immunization and sample collection. Three groups of mice (*n* = 4) were immunized with MCMV-RBD, MCMV-N, or MCMV-S-full at weeks 0, 4, and 8, and sera were collected at weeks 0, 2, 4, 6, 8, 10, and 12. (**D**) The SARS-CoV-2-specific IgG antibody titer was determined by indirect ELISA. The MCMV-RBD and MCMV-S-full immunized mice sera collected at different time points were serially diluted and tested using RBD-based indirect ELISA, and the MCMV-N immunized mice sera were tested using N-based indirect ELISA. (**E**) The endpoint dilution titer of MCMV-RBD/N/S-full immunized mice sera collected at week 12 was measured by indirect ELISA (cutoff: *P*/*N* > 2.5). (**F**) The NT_50_ of MCMV-RBD and MCMV-S-full immunized mice sera collected at different time points were determined using luciferase-tagged pseudovirus expressing Spike protein of the Wuhan-Hu-1 strain. (**G**) The NT_50_ of MCMV-RBD and MCMV-S-full immunized mice sera collected at different time points were determined using luciferase-tagged pseudovirus expressing SARS-CoV-2 Omicron variant (BA.2) Spike protein. (**H**) Comparison of the NT_50_ of MCMV-RBD immunized mice sera collected at different time points against pseudovirus expressing Spike protein of the Wuhan-Hu-1 strain and the BA.2 variant. (**I**) Comparison of the NT_50_ of MCMV-S-full immunized mice sera collected at different time points against pseudovirus expressing Spike protein of the Wuhan-Hu-1 strain and the BA.2 variant. Data are shown as mean ± SD. Significance was calculated using the two-tailed Student’s *t*-test for two groups. n.s., not significant; *P < 0.05; **P < 0.01; and ***P < 0.001.

Recombinant MCMV-S-full, MCMV-RBD, and MCMV-N were constructed using the same strategy as MCMV-Luc. All three genes were amplified from strain Wuhan-Hu-1 (GenBank: NC_045512), and the gene sequences have been codon optimized. All three genes (the intrinsic signal peptide of S-full was removed) were fused with the tissue plasminogen activator 1 (tPA-1) signal peptide, driven by the CMV promoter, and terminated with BGH-polyA; the expression cassettes were inserted into the IE2 locus of the MCMV genome (Fig. 2A). The expression of the three genes was confirmed by Western blot using an anti-RBD monoclonal mouse antibody or anti-N monoclonal rabbit antibody (Fig. 2B).

BALB/c mice (*n* = 4) were i.p. immunized at a dose of 10^7^ TCID_50_ per mouse with MCMV-S-full, MCMV-RBD, or MCMV-N at weeks 0, 4, and 8, and unimmunized mice (*n* = 2) were caged with the immunized mice in all three groups. Blood was collected at weeks 0, 2, 4, 6, 8, 10, and 12 (Fig. 2C). Recombinant RBD and N protein (both protein genes were amplified from strain Wuhan-Hu-1) were expressed and purified (Fig. S1A and D). Indirect ELISA assays were established to measure the level of antibody binding to SARS-CoV-2 RBD (Fig. S1E and F) or N protein (Fig. S1B and C). A pseudovirus neutralization assay was developed to measure the level of neutralizing antibodies against the SARS-CoV-2 original strain and a highly mutated Omicron variant (BA.2) (Fig. S2 to S4). Sera of mice immunized with MCMV-RBD and MCMV-S-full were evaluated by both RBD protein-based indirect ELISA and luciferase-tagged pseudovirus neutralization assays, and MCMV-N immunized mice sera were evaluated by N protein-based indirect ELISA. The result showed that mice of the three immunized groups developed detectable SARS-CoV-2-specific antibodies 2 weeks after initial immunization; the level of antibodies increased 4 weeks post-immunization and was significantly elevated by booster immunization (Fig. 2D). The average endpoint dilution titer of MCMV-S-full-, MCMV-RBD-, and MCMV-N-immunized mice sera collected at week 12 was around 6,250, 31,250, and 156,250, respectively (Fig. 2E).

The Omicron variant (BA.2) is a highly mutated virus strain containing many vaccine escape mutations (such as K417N and D614G) and vaccine weakening mutations (such as N501Y and Q493R) in the Spike protein (Fig. S3A) (27, 28). To examine if the antibodies elicited by CMV-vectored vaccines expressing the S/RBD protein of the SARS-CoV-2 strain circulating early in the pandemic still can neutralize the BA.2 variant, luciferase-tagged pseudoviruses expressing the Spike protein of Wuhan-Hu-1 strain or BA.2 variant were constructed, and a pseudovirus neutralization assay was performed to evaluate the neutralizing ability of MCMV-S-full- and MCMV-RBD-immunized mice sera. The results showed that neutralizing antibodies against both the Wuhan-Hu-1 strain and the BA.2 variant could be detected as early as 2 weeks post-initial immunization of MCMV-RBD, and the titer increased 4 weeks post-immunization. However, only low neutralizing antibodies against the Wuhan-Hu-1 could be detected 2 and 4 weeks post-initial immunization of MCMV-S-full. At the same time, almost no neutralizing antibodies against the BA.2 variant were detected in MCMV-S-full-immunized mice sera collected at weeks 2 and 4 (Fig. 2F and G). The results also showed that first booter immunization could significantly elevate the neutralizing antibodies’ titer against both the Wuhan-Hu-1 strain and the BA.2 variant when immunized with MCMV-RBD or MCMV-S-full (week 6). At the same time, the level of neutralizing antibodies did not increase significantly after the second booster immunization (Fig. 2F and G). MCMV-RBD elicited a higher level of neutralizing antibodies against both the Wuhan-Hu-1 strain and the BA.2 variant than MCMV-S-full (Fig. 2F and G). Moreover, the neutralizing ability of MCMV-RBD- and MCMV-S-full-immunized sera collected at different time points against the Wuhan-Hu-1 strain and the BA.2 variant was compared, and the results showed the neutralizing antibody titers against the BA.2 variant were significantly lower than the neutralizing antibody titers against the Wuhan-Hu-1 strain (Fig. 2H and I). Mutations on the Spike protein of the BA.2 variant might provide resistance against vaccine-induced immune responses.

### CMV viral vectors were not transmitted from immunized mice to unimmunized mice

**Fig 3:**
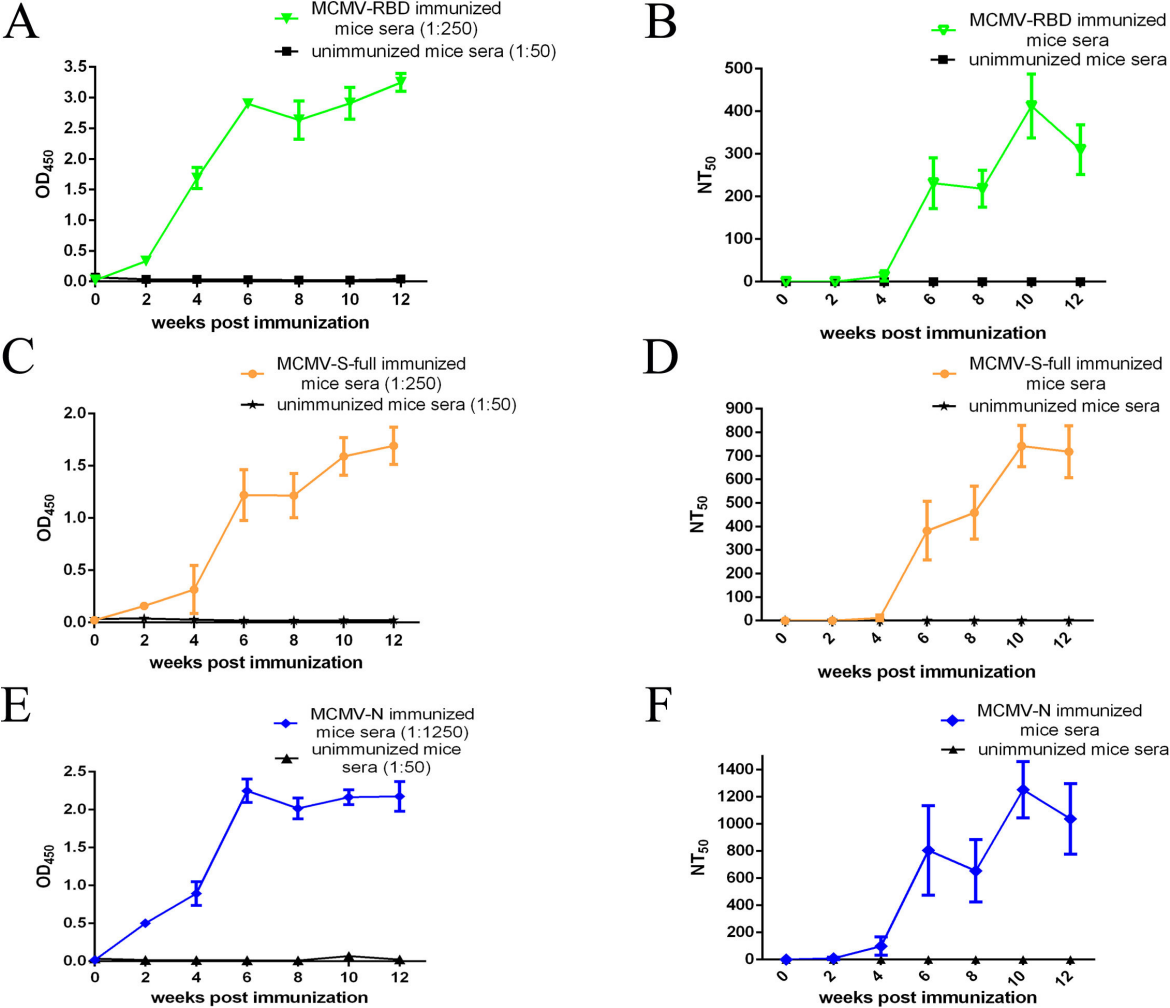
Evaluation of unimmunized mice sera antibodies caged with CMV-vectored vaccine-immunized mice. (**A**) Measure of RBD-binding IgG antibodies in the MCMV-RBD immunized or unimmunized mice sera. (**B**) Measure of neutralizing antibodies specific to MCMV in the MCMV-RBD immunized or unimmunized mice sera. (**C**) Measure of RBD-binding IgG antibodies in the MCMV-S-full immunized or unimmunized mice sera. (**D**) Measure of neutralizing antibodies specific to MCMV in the MCMV-S-full immunized or unimmunized mice sera. (**E**) Measure of RBD-binding IgG antibodies in the MCMV-N immunized or unimmunized mice sera. (**F**) Measure of neutralizing antibodies specific to MCMV in the MCMV-N immunized or unimmunized mice sera.

MCMV caused transient systematic infection in mice, especially luciferase signal could be detected at the natural exit sites (salivary glands) during the late infection phase. To investigate the transmissibility of the CMV-vectored vaccine, two unimmunized mice were caged in the same cage as the immunized mice in all three groups. Blood of unimmunized mice was collected at weeks 0, 2, 4, 6, 8, 10, and 12, and SARS-CoV-2-specific antibodies were tested by indirect ELISA assay. SARS-CoV-2-specific antibodies could be detected in the immunized mice sera collected at different time points in all three groups. In contrast, no SARS-CoV-2-specific antibody could be detected in the unimmunized mice sera caged with the immunized mice. (Fig. 3A, C, and E)

Luciferase-tagged MCMV was used to establish a neutralization assay to evaluate the immune response against the CMV vector, and the neutralizing antibodies of unimmunized and immunized mice sera were evaluated. In all three immunized groups, no or low levels of neutralizing antibodies against MCMV were detected at 2 or 4 weeks post primary immunization, and the levels of neutralizing antibodies could be detected after the first booster immunization and were further elevated after the second booster immunization. In contrast, no neutralizing antibodies against MCMV could be detected in the serum samples collected at different time points in all three groups of unimmunized mice caged with immunized mice (Fig. 3B, D, and F).

### Pre-existing immunity against CMV weakened the efficacy of CMV-vectored vaccine

**Fig 4:**
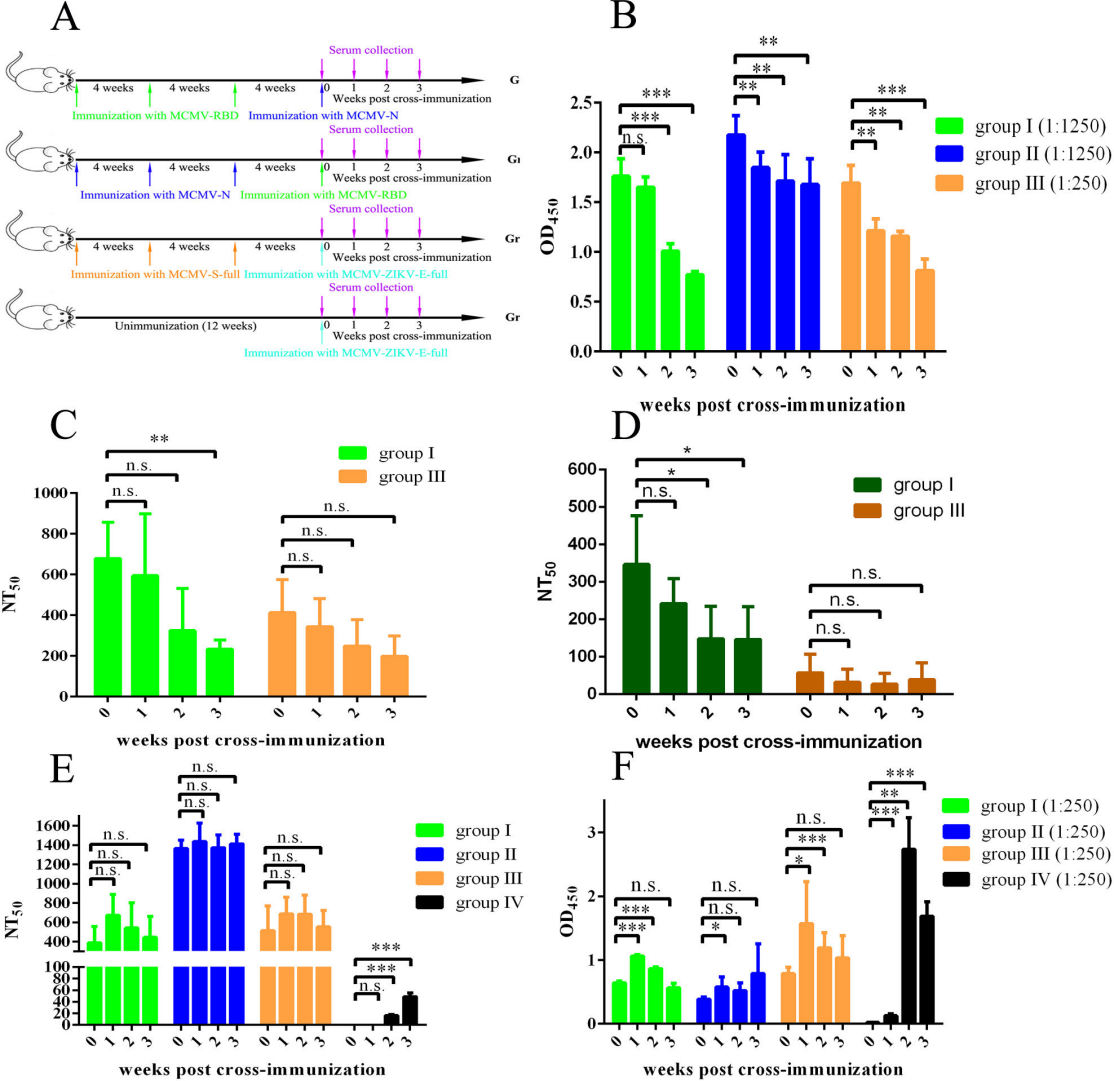
The impacts of pre-existing immunity on the efficacy of CMV-vectored vaccines. (**A**) Schematic diagram of cross-immunization and sample collection. Four groups of mice with/without pre-existing immunity were cross-immunized with MCMV-RBD, MCMV-N, or MCMV-Zika-E-full, and sera were collected at weeks 0, 1, 2, and 3. (**B**) Evaluation of RBD- or N-binding IgG antibodies in the group I/II/III mice sera pre- or post-cross-immunization. RBD-binding IgG antibodies in groups I and III mice sera were detected by RBD-based indirect ELISA, and N-binding IgG antibodies in the group II mice sera were detected by N protein-based indirect ELISA. (**C**) Evaluation of neutralizing antibodies against SARS-CoV-2 Wuhan-Hu-1 strain in groups I and III mice sera pre- or post-cross-immunization. (**D**) Evaluation of neutralizing antibodies against SARS-CoV-2 Omicron variant (BA.2) in groups I and III mice sera pre- or post-cross-immunization. (**E**) Evaluation of neutralizing antibodies against MCMV viral vector in groups I, II, III, and IV mice sera pre- or post-cross-immunization. (**F**) Evaluation of SARS-CoV-2- or ZIKV-specific antibodies in groups I, II, III, and IV mice sera pre- or post-cross-immunization. N protein-binding antibodies in group I mice sera were detected by N protein-based indirect ELISA; RBD-binding antibodies in group II mice sera were detected by RBD-based indirect ELISA; ZIKV E protein-binding antibodies in groups III and IV mice sera were detected by E-DIII-based indirect ELISA.

To investigate the influences of pre-existing immunity against the CMV vector on the efficacy of the CMV-vectored vaccine, the three groups of mice were cross-immunized with different CMV-vectored vaccines after three-dose immunization. The group of MCMV-RBD immunized mice was cross-immunized with MCMV-N (group I), and MCMV-N immunized mice were cross-immunized with MCMV-RBD (group II). The group of MCMV-S-full immunized mice (group III) and unimmunized mice (group IV) were immunized with an MCMV-vectored vaccine expressing full-length E protein of Zika virus (MCMV-Zika-E-full), and the gene of E protein was amplified from Zika virus strain PRVABC59 (GenBank: KU501215). Blood was collected at 0-, 1-, 2-, and 3-weeks post-cross-immunization (Fig. 4A; Fig. S5). The DIII domain of Zika virus E protein (296–403 aa) was recombinant expressed and used to establish an indirect ELISA to measure Zika virus-specific antibodies (Fig. S6). The level of SARS-CoV-2-specific antibodies was measured with RBD protein-based indirect ELISA (group I and group III) or N protein-based indirect ELISA (group II), and the results showed that the pre-existing SARS-CoV-2-specific antibodies decreased significantly when cross-immunized with a different MCMV-vectored vaccine (Fig. 4B). The levels of neutralizing antibodies against SARS-CoV-2 were evaluated with luciferase-tagged pseudovirus neutralization assays. The levels of neutralizing antibodies against both the Wuhan-Hu-1 strain and the BA.2 variant were significantly decreased after cross-immunization (group I), while that of group III decreased insignificantly (Fig. 4C and D). Robust neutralizing antibodies against MCMV were elicited by immunization of three doses of CMV-vectored vaccines before cross-immunization, and the level of neutralizing antibodies showed no significant difference before and after cross-immunization (groups I, II, and III); neutralizing antibodies specific to MCMV could be detected 2 weeks post-immunization in group IV (Fig. 4E). When different CMV-vectored vaccines were applied to the mice with pre-existing immunity against MCMV, antibodies could be elicited as early as 1-week post-immunization. However, the level of antibodies showed no significant difference between sera collected pre-cross immunization and 3 weeks post-cross immunization (Fig. 4F, groups I, II, and III). In comparison, higher levels of ZIKV-specific antibodies were elicited and maintained significant differences post-immunization in group IV (Fig. 4F). These results indicated that when sequential immunization of an individual with different CMV-vectored vaccines occurs, the efficacy of the latter vaccine might be weakened by the immunity elicited by the first-immunized vaccine. Pre-existing immunity against CMV might limit viral replication and compromise vaccine efficacy when cross-immunized with different CMV-vectored vaccines.

## DISCUSSION

Safety is one of the most critical aspects of developing viral vectors. Viral vectors should be able to efficiently deliver the target genes into host cells and enable their expression *in vivo*. Two strategies are commonly used to ensure the safety of virus-vectored vaccines. One strategy is to construct replication-defective viruses by genetic engineering. These viral vectors can usually enter and deliver the target genes into the host cells, but no infectious progeny virions will be generated to cause further infection. Another strategy is to develop attenuated viruses as viral vectors; these viral vectors can infect host cells, deliver target genes into host cells, and generate infectious progeny virions. However, these infections usually do not cause severe clinical symptoms. In the case of CMV, a replication-defective virus has been constructed and is under evaluation in clinical trials for safety and immunogenicity. Three doses administered to CMV-seronegative recipients showed no serious adverse events and robust immune responses in Phase I clinical trials (29, 30). Fibroblast cell-adapted CMV strains Towne and AD169 were attenuated and were safe and well-tolerated when tested in clinical trials (31, 32). CMV should be reasonably safe as a viral vector.

When CMV was cultured *in vitro*, the titer of infectious CMV virion in supernatant usually peaks at 10^7^ PFU/mL. Moreover, CMV is easily inactivated in freeze-thaw cycles, and special methods must be used to retain its infectious ability. The attenuated CMV virus retains its replication ability *in vivo* and can induce robust expression of the target gene. The attenuated vector vaccine usually shows stronger immunogenicity and requires fewer and lower doses to elicit robust immune responses. However, CMV is a strict species-specific virus, and its dissemination feature *in vivo* still needs to be better understood. Currently, several primate and nonprimate animal models are available for CMV vaccine and pathogenesis research (33, 34). In this study, a luciferase gene expression cassette was constructed into the genome of MCMV to generate a reporter virus, and using the reporter virus to infect mice provided an ideal model to quantitatively track the dissemination of CMV *in vivo* (35, 36). The bioluminescent signals were first detected at the injection site and spread quickly to the whole enterocoelia; this indicated that CMV could infect multiple organs and induce robust expression of the foreign gene. The bioluminescent signals could be detected as early as 2 days post-injection and faded to undetectable levels more than 17 days post-injection. In the evaluation of an mRNA vaccine, the bioluminescent signals could be detected from 6 to 48 h post-injection, and the liver was the most abundant foreign gene-expressing tissue (37). A relatively long time for the expression of foreign genes and a wide range of cell tropism using CMV as a viral vector might help to elicit robust immune responses.

In the late phase of infection, CMV starts replicating in the salivary gland, which is the natural exit site for CMV. Whether the virus of the CMV-vectored vaccine can be transmitted to unimmunized individuals is a crucial safety concern of the CMV viral vector. In this study, unimmunized mice were caged with mice immunized with three different CMV-vectored vaccines, and blood was collected and tested for SARS-CoV-2-specific antibodies using indirect ELISA. SARS-CoV-2-specific antibodies were ready to be detected in the immunized mice sera of all three groups as early as 2 weeks post-immunization at the dilution of 1:250. However, no SARS-CoV-2-specific antibodies could be detected in all the unimmunized mice sera collected at any time points, even at the dilution of 1:50. Furthermore, neutralizing antibodies specific to MCMV could be detected in all the immunized mice sera, and no neutralizing antibodies could be detected in the unimmunized mice caged with immunized mice. These results showed that the viruses of the CMV-vectored vaccine are not transmittable and might not cause uncontrolled transmission in the population.

Accumulated data showed that CMV-vectored vaccines could elicit both humoral and cellular immune responses in different animal models and provide good protection against challenges with virulent pathogens (14, 38 – 41). In the case of SARS-CoV-2, antibody titer (especially neutralizing antibodies) was proven to be well-correlated with protection against SARS-CoV-2 infection in non-human primates and a large number of human samples (42 – 44). The role of cellular immunity in protecting against SARS-CoV-2 infection is sometimes controversial in different research and still needs clarification (45, 46). In this study, CMV-vectored SARS-CoV-2 vaccines expressing the major structural proteins (RBD, Spike, or Nucleocapsid protein) of SARS-CoV-2 were constructed and evaluated for immunogenicity. Spike protein is the major membrane protein, and the RBD domain binds directly to the ACE2 receptor; both could elicit neutralizing antibodies to block the infection of SARS-CoV-2. The S protein or RBD domain has been used as the major immunogen in the currently authorized COVID-19 vaccines or COVID-19 vaccine candidates for clinical and pre-clinical development. In this study, vaccines based on full-length Spike or RBD domain were constructed and evaluated on the same viral vector platform. MCMV-RBD or MCMV-S-full infected cell lysates were incubated with the same anti-RBD monoclonal antibody and analyzed by western blot; stronger specific bioluminescence signals were obtained in the lane loaded with MCMV-RBD-infected cell lysate (Fig. 2B); it indicated that the expression level of RBD was much higher than that of full-length Spike using CMV as the viral vector. S-full protein is a high-molecular-weight membrane protein, and it is much more difficult to express in cells. Furthermore, MCMV-RBD and MCMV-S-full were used to immunize BALB/c mice with the same strategy. Compared with MCMV-RBD immunized group, MCMV-S-full elicited a much higher level of neutralizing antibodies specific to MCMV (Fig. 3B and D). However, MCMV-S-full elicited lower RBD-specific antibodies and neutralizing antibodies against both the Wuhan-Hu-1 strain and the Omicron variant (BA.2) than MCMV-RBD. (Fig. 2D through I) These results indicated that vaccines using RBD protein as the major immunogen showed stronger immunogenicity than vaccines using full-length spike protein. The study also showed that neutralizing antibodies elicited by vaccines based on the original strain from Wuhan (Wuhan-Hu-1) still can neutralize the highly mutated Omicron variants. However, the level of neutralizing antibodies against the BA.2 variant was much lower than that against the Wuhan-Hu-1 strain; it indicated that mutations on the Spike protein of BA.2 facilitate the variant escape from vaccine-mediated immunity. The rational design of a vaccine with enhanced immunogenicity might also be an effective way to cope with the numerous variants of SARS-CoV-2 besides the development of multivalent SARS-CoV-2 vaccines.

Since the first case of COVID-19 was identified in December 2019, COVID-19 spread quickly to become a global pandemic, and the pandemic is still ongoing worldwide. One of the most critical factors contributing to the long-term pandemic is the rapid evolution of the SARS-CoV-2 variants. Currently, most authorized or candidate SARS-CoV-2 vaccines use spike protein as the major immunogen and focus on eliciting neutralizing antibodies that could block the infection of the virus. However, the Spike protein gene is prone to mutation, and thousands of mutations in the Spike protein gene have been identified (47). Mutations occurring in the S gene region might enhance the transmissibility and compromise the protective immunity elicited by vaccines or natural infections (48, 49). Nucleocapsid protein is more conservative and has less mutation than the Spike protein, and some new variant viruses that can partially escape humoral response do not significantly escape T-cell response (50). The Nucleocapsid protein is one of the main structural proteins that can trigger T-cell responses in humans, and a Nucleocapsid-based vaccine was proven to elicit Spike-independent protective immunity against SARS-CoV-2 (51). In this study, a CMV-vectored vaccine expressing the Nucleocapsid protein was constructed and evaluated; the vaccine was able to induce high-level expression of Nucleocapsid protein *in vitro* and elicited robust humoral responses in mice. These works might provide an optimal choice for developing broad-spectrum SARS-CoV-2 vaccines.

Primary infection of human CMV will establish a lifelong latent infection in healthy individuals, and the latent virus will occasionally reactivate to boost host immunity and maintain high frequencies of effector T cells in the circulation (52). However, no latent infection or virus reactivation was observed in mice following primary infection with MCMV-Luc. When immunizing BALB/c mice with CMV-vectored vaccines, single-dose immunization was able to elicit SARS-CoV-2-specific antibodies; however, the level of antibodies could be elevated significantly by booster immunization in all three groups. Primary immunization of MCMV-RBD elicited low neutralizing antibodies specific to SARS-CoV-2 measured with a pseudovirus neutralization assay. In contrast, almost no neutralizing antibody could be detected after primary immunization of MCMV-S-full. Booster immunization elicited higher levels of neutralizing antibodies specific to SARS-CoV-2. These results indicated that at least two doses of CMV-vectored vaccine are essential to elicit strong immune responses to provide better protection.

Viral vectors are powerful tools to deliver heterologous genes *in vivo*. Multiple viral vectors, including adenovirus, vesicular stomatitis virus, and vaccinia virus, have been evaluated in vaccine development and showed great promise (5, 53 – 55). However, pre-existing immunity against the viral vectors in the host might compromise the immunogenicity and protection conferred by other vaccines based on the same viral vectors (8, 56). CMV-based viral vector is expected to circumvent pre-existing immunity since secondary infection, mixed infection, or virus reactivation could occur in the presence of pre-existing immunity against CMV (57, 58). In this study, various CMV-vectored vaccines based on the same virus strain were constructed and used to examine the influences of pre-existing immunity on vaccine efficacy. The results of the neutralization assay using MCMV-Luc showed that at least two doses of CMV-vectored vaccines were required to elicit neutralizing antibodies against MCMV. The CMV-specific immunity peaked after the third dose (Fig. 3B, D, and F). In contrast, there was no significant increase in the level of neutralizing antibodies after the fourth dose (Fig. 4E). The study showed that booster immunization could significantly elevate the level of SARS-CoV-2-specific antibodies even in the presence of CMV-specific immunity. When mice receiving three doses of CMV-vectored vaccines were cross-immunized with CMV-vectored vaccines expressing different heterogenous proteins, antibodies specific to the second protein could be detected much lower than in no pre-existing immunity groups (Fig. 4F). These results indicated that CMV-vectored vaccines were still immunogenic in the presence of pre-existing immunity. However, the pre-existing immunity could partly compromise the immunogenicity of CMV-vectored vaccines.

In conclusion, the study showed CMV could cause transient systemic infection and drive foreign gene expression *in vivo*. CMV-vectored COVID vaccines retain the ability to induce high levels of binding antibodies and neutralizing antibodies against both the original strain and the BA.2 variant, and CMV viral vector would not cause unexpected transmission. CMV is a promising viral vector for vaccine development.

## Data Availability

The data sets used and/or analyzed during the current study are available from the corresponding author upon reasonable request.

## SUPPLEMENTAL MATERIAL

Fig. S1 spectrum.02463-23-s0001.tif Establishment of SARS-CoV-2 receptor-binding domain (RBD) and nucleocapsid (N) based indirect ELISA.

Fig. S2 spectrum.02463-23-s0002.tif Construction of HEK 293T clonal cell lines constitutively expressing human ACE2 (293T-ACE2).

Fig. S3 spectrum.02463-23-s0003.tif Construction of reporter SARS-CoV-2 pseudovirus expressing spike protein of SARS-CoV-2 Wuhan Hu-1 strain or omicron variant BA.2.

Fig. S4 spectrum.02463-23-s0004.tif Comparison of the signals generated by transduction of EGFP-tagged SARS-CoV-2 pseudovirus and luciferase-tagged SARS-CoV-2 pseudovirus.

Fig. S5 spectrum.02463-23-s0005.tif Construction and verification of MCMV-ZIKV-E-full.

Fig. S6 spectrum.02463-23-s0006.tif Establishment of ZIKV E protein domain III (E-DIII) based indirect ELISA.

Supplemental legends spectrum.02463-23-s0007.docx Legends for Fig. S1 to S6.

ASM does not own the copyrights to Supplemental Material that may be linked to, or accessed through, an article. The authors have granted ASM a non-exclusive, world-wide license to publish the Supplemental Material files. Please contact the corresponding author directly for reuse.
