# Supplementary figures and images for: Cytomegalovirus-vectored COVID-19 vaccines elicit neutralizing antibodies against the SARS-CoV-2 Omicron variant (BA.2) in mice
Source: Microbiol Spectr. 2023 Nov 16;11(6):e02463-23. doi: 10.1128/spectrum.02463-23 (PMC10883801; doi:10.1128/spectrum.02463-23)

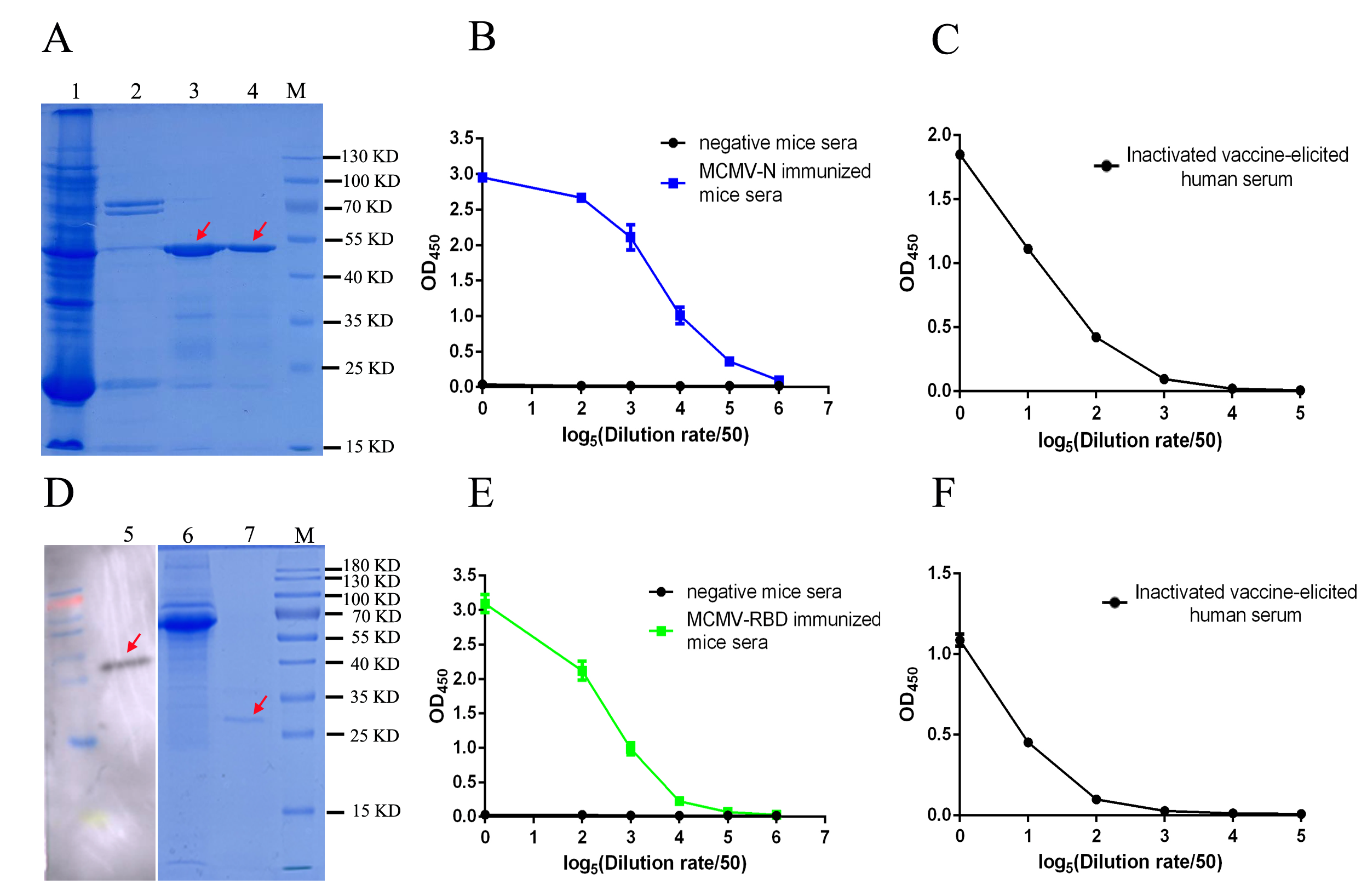

Supplement: Fig. S1 — Establishment of SARS-CoV-2 receptor-binding domain (RBD) and nucleocapsid (N) based indirect ELISA. [file spectrum.02463-23-s0001.tif]

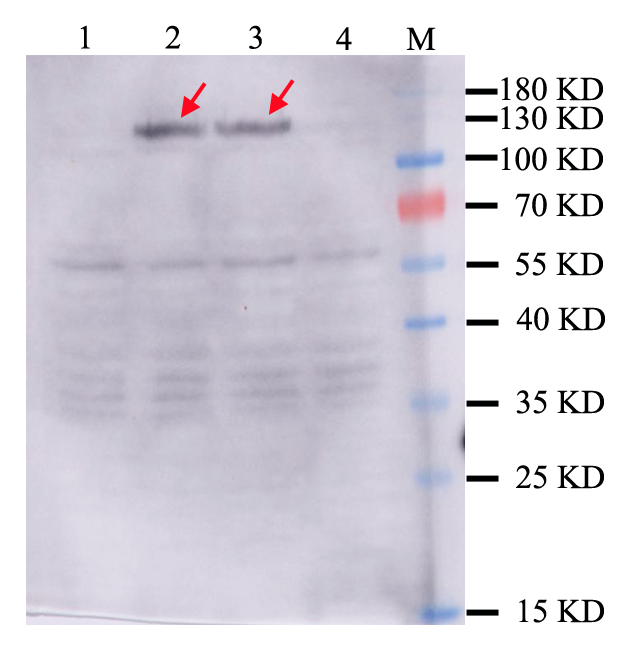

Supplement: Fig. S2 — Construction of HEK 293T clonal cell lines constitutively expressing human ACE2 (293T-ACE2). [file spectrum.02463-23-s0002.tif]

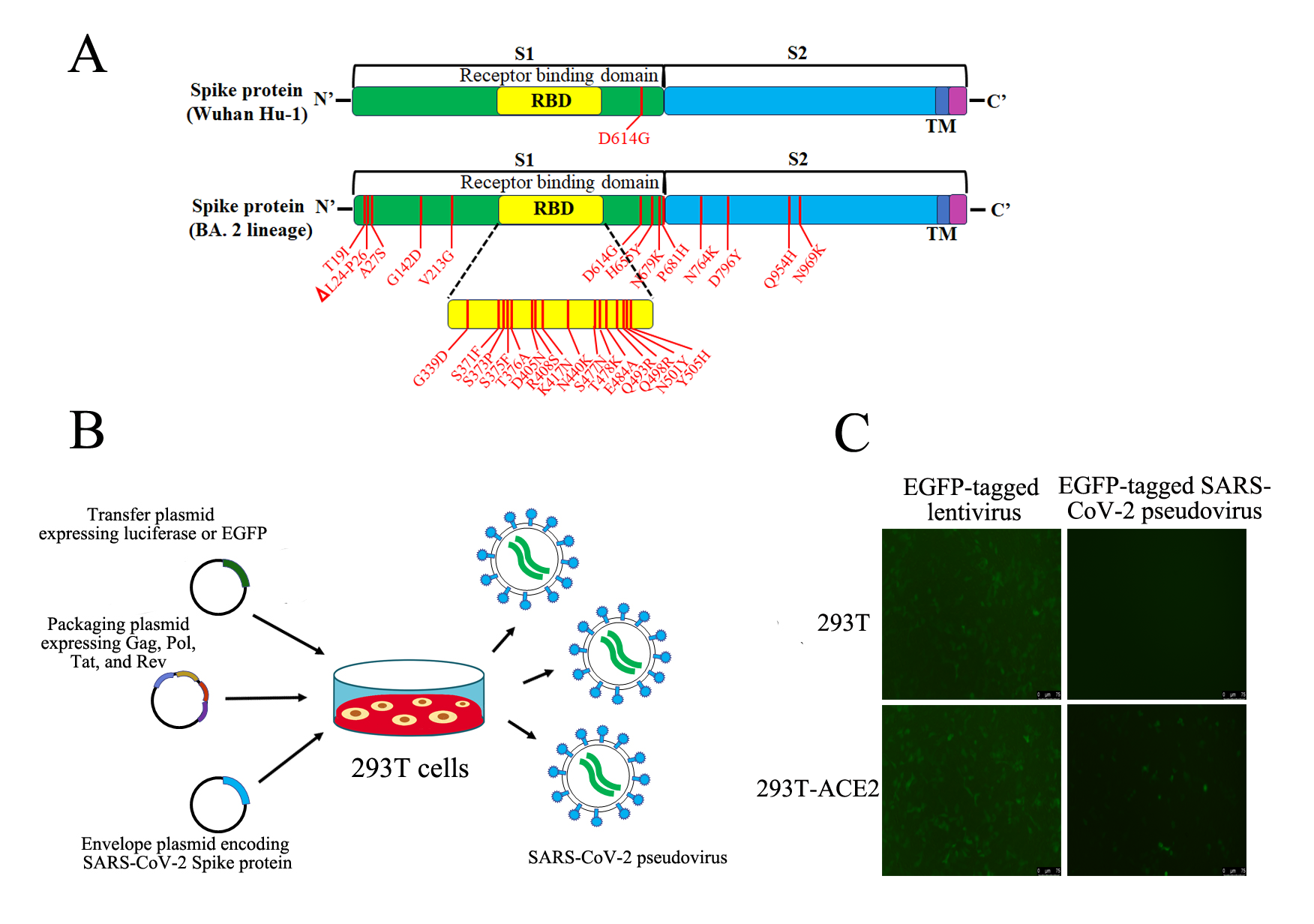

Supplement: Fig. S3 — Construction of reporter SARS-CoV-2 pseudovirus expressing spike protein of SARS-CoV-2 Wuhan Hu-1 strain or omicron variant BA.2. [file spectrum.02463-23-s0003.tif]

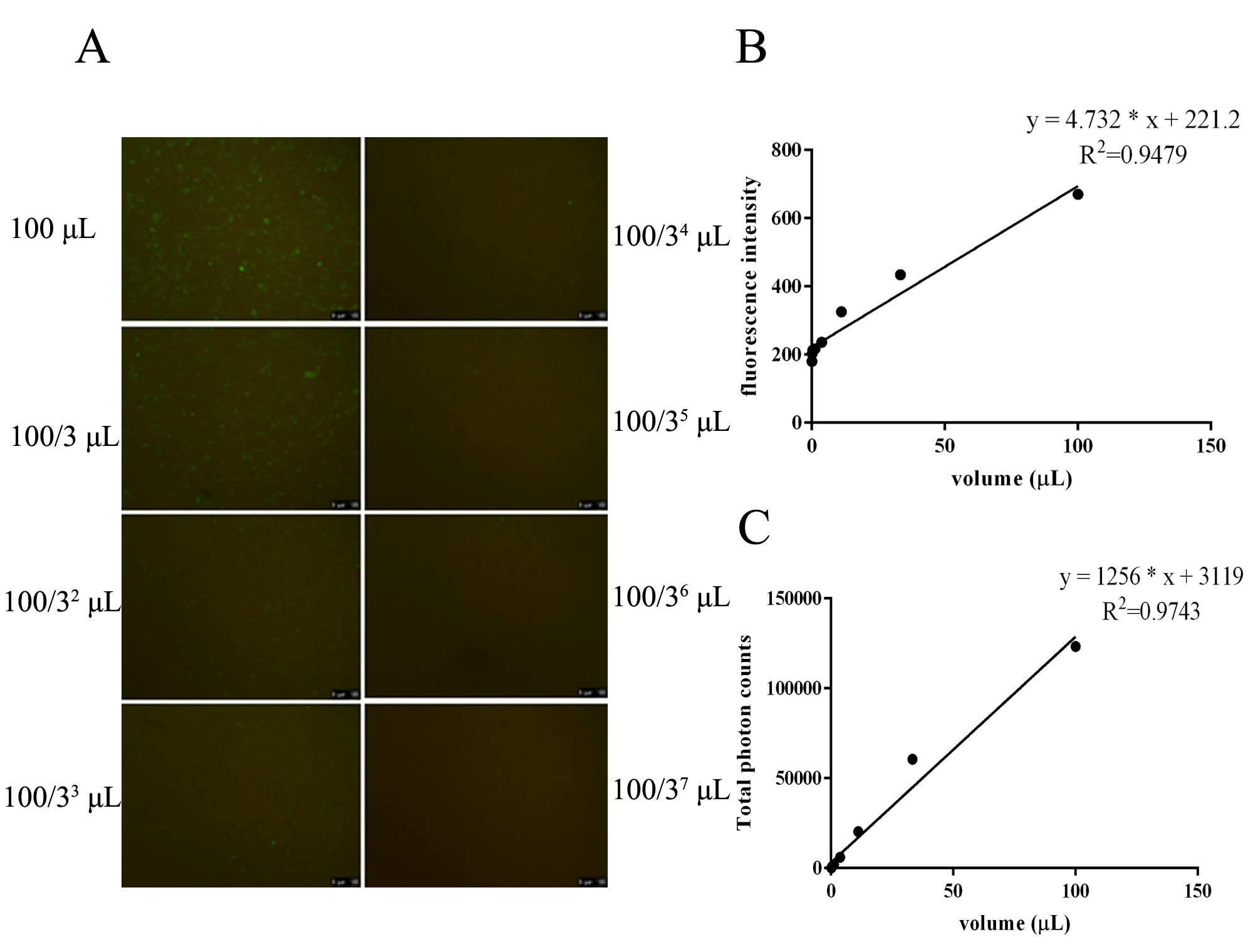

Supplement: Fig. S4 — Comparison of the signals generated by transduction of EGFP-tagged SARS-CoV-2 pseudovirus and luciferase-tagged SARS-CoV-2 pseudovirus. [file spectrum.02463-23-s0004.tif]

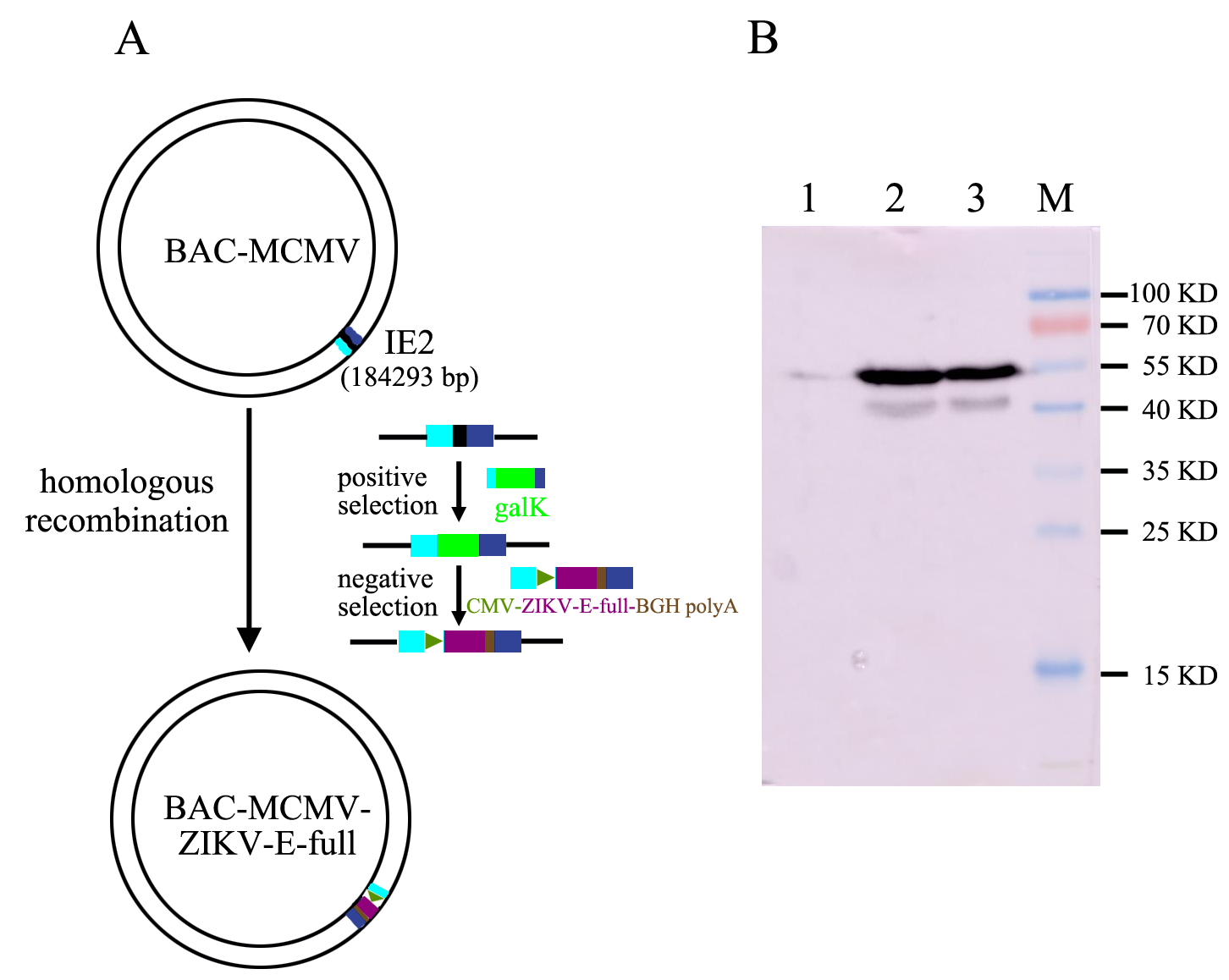

Supplement: Fig. S5 — Construction and verification of MCMV-ZIKV-E-full. [file spectrum.02463-23-s0005.tif]

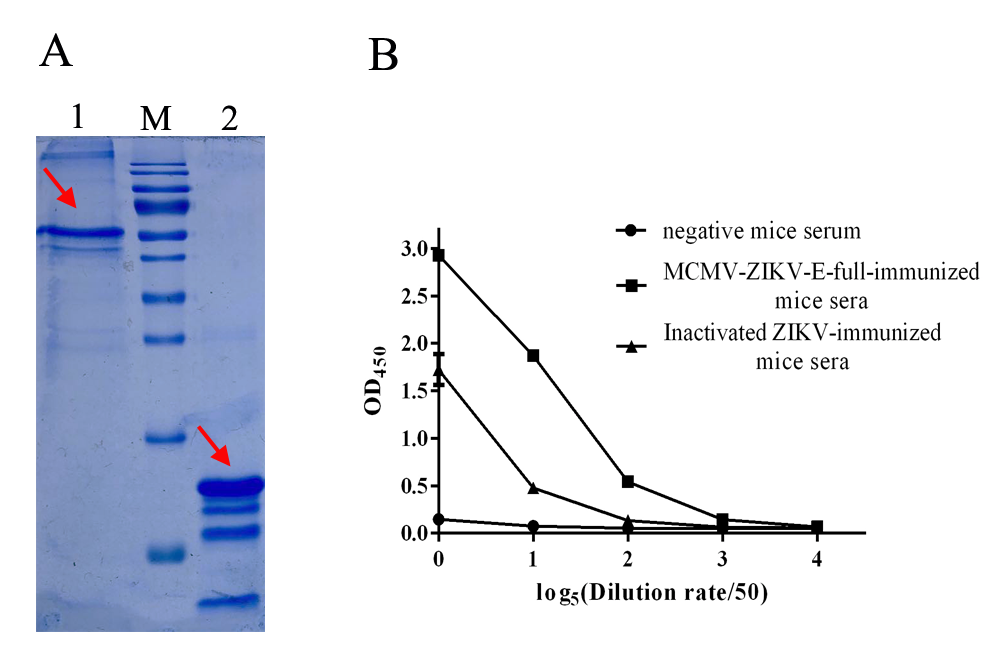

Supplement: Fig. S6 — Establishment of ZIKV E protein domain III (E-DIII) based indirect ELISA. [file spectrum.02463-23-s0006.tif]
