# Supplementary material for: Cytomegalovirus-vectored COVID-19 vaccines elicit neutralizing antibodies against the SARS-CoV-2 Omicron variant (BA.2) in mice
Source: Microbiol Spectr. 2023 Nov 16;11(6):e02463-23. doi: 10.1128/spectrum.02463-23 (PMC10883801; doi:10.1128/spectrum.02463-23)
Supplement: Supplemental legends — Legends for Fig. S1 to S6. [file spectrum.02463-23-s0007.docx]

Supplementary Materials for

**Cytomegalovirus-vectored COVID-19 vaccines elicit neutralizing antibodies against the SARS-CoV-2 Omicron variant (BA.2) in mice**

Jian Liu^1, *^, Dabbu Kumar Jaijyan^2^, Yanling Chen^1^, Changcan Feng^1^, Shaomin Yang^3^, Zhenglong Xu^1^, Nichun Zhan^1^, Congming Hong^4^, Shuxuan Li^4^, Tong Cheng^4^, Hua Zhu^2, *^

* Correspondence: [lj1861@mnnu.edu.com](mailto:lj1861@mnnu.edu.com) (J. L.); [Zhuhu@njms.rutgers.edu](mailto:Zhuhu@njms.rutgers.edu) (H. Z.)

**Fig. S1 Establishment of SARS-CoV-2 receptor-binding domain (RBD) and nucleocapsid (N) based indirect ELISA.**

The gene of SARS-CoV-2 nucleocapsid (N) was cloned into the pET-28a (+) vector and expressed in bacterial strain BL21 (DE3). The receptor-binding domain (RBD) of the SARS-CoV-2 spike protein (319-541 aa) was secretory expressed using the baculovirus-insect cell expression system as a fusion protein containing the N-terminal gp67s signal peptide and a C-terminal 6$\times$his tag. Both recombinant RBD and N proteins were purified using nickel-based affinity chromatography. (Fig. S1A, lanes 3&4; Fig. S1D, lanes 5&7) Purified recombinant RBD and N protein were used to establish indirect ELISA assays to measure the level of SARS-CoV-2 specific antibodies in serum. MCMV-RBD or MCMV-N immunized mice serum, unimmunized mice serum, and human serum collected after three doses of inactivated SARS-CoV-2 vaccine vaccination were used to test the specificity and sensitivity of the RBD or N protein-based indirect ELISA assays. (Fig. S1B, S1C, S1E, S1F). Human serum was taken from one of the authors (J. L.), and written informed consent was obtained.

**Fig. S2 Construction of HEK 293T clonal cell lines constitutively expressing human ACE2 (293T-ACE2)**

293T-ACE2 cell line was constructed using a lentiviral vector. Human full-length ACE2 gene (GenBank: NM_021804.2) fused with a 3$\times$flag tag sequence was constructed into the transfer plasmid (pLVX-ACE2-3$\times$flag). The recombinant transfer plasmid (pLVX-ACE2-3$\times$flag), packaging plasmid (psPAX2), and envelope plasmid (pMD2.G) were co-transfected into HEK 293T cells. The supernatant containing lentivirus was collected 72 h post-transfection and used to transduce HEK 293T cells, and 3 mg/mL Blasticidin was added to select antibiotics-resistance cells 48 h post-transduction. Monoclonal cells were obtained by the limiting-dilution method and re-expanded into large populations. Expanded cell clones were lysed and analyzed by Western Blot with an anti-flag tag monoclonal antibody (#66008-4-Ig; Proteintech, Wuhan, China), and two clonal cell lines stably expressing ACE2 were obtained (lane 2 & 3).

**Fig. S3 Construction of reporter SARS-CoV-2 pseudovirus expressing spike protein of SARS-CoV-2 Wuhan Hu-1 strain or omicron variant BA.2**

(A) Schematic diagram of the Spike protein of the Wuhan Hu-1 strain and Omicron variant (BA.2). The spike protein of the BA.2 variant is characterized by many mutations and deletions within the Spike coding sequence. The Spike gene was also codon-optimized, and the last 19 aa were removed.

(B) Reporter pseudotyping lentiviral particles were constructed by co-transfecting 293T cells with a lentiviral backbone encoding a reporter protein (EGFP or firefly luciferase; pLVX-EGFP/ luciferase), a plasmid expressing codon-optimized SARS-CoV-2 spike protein (pLV-Spike), and a plasmid encoding the other proteins necessary for virion formation (psPAX2).

(C) Verification of EGFP-tagged SARS-CoV-2 pseudovirus. EGFP-tagged lentivirus pseudotyped with vesicular stomatitis virus envelope glycoprotein (VSV-G) was able to transduce both 293T and 293T-ACE2 cells. In contrast, EGFP-tagged SARS-CoV-2 pseudovirus pseudotyped with Spike protein could only transduce 293T-ACE2 cells. Compared with EGFP-tagged lentivirus, the transduction efficiency and fluorescent signal are much lower when using EGFP-tagged SARS-CoV-2 pseudovirus to transduce 293T-ACE2 cells.

**Fig. S4 Comparison of the signals generated by transduction of EGFP-tagged SARS-CoV-2 pseudovirus and luciferase-tagged SARS-CoV-2 pseudovirus**

1. EGFP-tagged SARS-CoV-2 pseudovirus was 3-fold serially diluted and used to transduce 293T-ACE2 cells plated in transparent flat 96-well plates (100 μL/well). 4 days post-transduction, fluorescence signals of cells plated in transparent 96-well plates were recorded by a fluorescence microscope (Leica).
2. EGFP-tagged SARS-CoV-2 pseudovirus was 3-fold serially diluted and used to transduce 293T-ACE2 cells plated in black flat 96-well plates (100 μL/well). 4 days post-transduction, cells were lysed, and the fluorescent signals were collected with a SparkControl Magellan plate reader. The correlation between fluorescent signals and the volume of pseudovirus was analyzed.
3. Luciferase-tagged SARS-CoV-2 pseudovirus was 3-fold serially diluted and used to transduce 293T-ACE2 cells plated in white flat 96-well plates (100 μL/well). 4 days post-transduction, the bioluminescent signals were collected with a SparkControl Magellan plate reader. The correlation between bioluminescent signals and the volume of pseudovirus was analyzed.

**Fig. S5 Construction and verification of MCMV-ZIKV-E-full**

1. Schematic diagram of the construction of MCMV-ZIKV-E-full. The expression cassette of the ZIKV full-length E protein gene (PRVABC59 strain; GenBank: KU501215) was inserted into the MCMV IE2 locus using the modified DH10B strain SW102 and a galK positive/counterselection cassette. (Warming, Costantino et al. 2005)
2. Verified the ZIKV full-length E protein expression using Western Blot. Uninfected NIH-3T3 cell lysates (lane 1) and MCMV-ZIKV-E-full infected NIH-3T3 cell lysates (lane 2&3) were immunoblotted with a homemade monoclonal antibody recognizing ZIKV E protein.

**Fig. S6 Establishment of ZIKV E protein domain III (E-DIII) based indirect ELISA.**

1. Expression and purification of ZIKV E ectodomain and E-DIII protein. E protein's ectodomain (1-455 aa) and DIII domain (296-403 aa) were expressed in *E.coli* as a fusion protein containing an N-terminal His_6_ tag and purified by Nickel affinity chromatography. Lane 1: purified E ectodomain; M: protein marker; Lane 2: purified E-DIII.
2. Evaluation of ZIKV E-DIII based indirect ELISA. Purified E-DIII protein was coated onto 96-well ELISA plates (100 ng/well), and serial diluted inactivated ZIKV-immunized, MCMV-ZIKV-E-full-immunized, and negative mice sera were used to evaluate the specificity and sensitivity of ZIKV E-DIII based indirect ELISA.
